# Innovative computational approaches shed light on genetic mechanisms underlying cognitive impairment among children born extremely preterm

**DOI:** 10.1186/s11689-022-09429-x

**Published:** 2022-03-03

**Authors:** Weifang Liu, Quan Sun, Le Huang, Arjun Bhattacharya, Geoffery W. Wang, Xianming Tan, Karl C. K. Kuban, Robert M. Joseph, T. Michael O’Shea, Rebecca C. Fry, Yun Li, Hudson P. Santos

**Affiliations:** 1 Department of Biostatistics, University of North Carolina at Chapel Hill, Chapel Hill, NC USA; 2 Curriculum in Bioinformatics and Computational Biology, University of North Carolina at Chapel Hill, Chapel Hill, NC USA; 3 Department of Pathology and Laboratory Medicine, David Geffen School of Medicine, University of California, Los Angeles, CA USA; 4 Lineberger Comprehensive Cancer Center, University of North Carolina at Chapel Hill, Chapel Hill, NC USA; 5 Department of Pediatrics, Boston University, Boston, MA USA; 6 Department of Anatomy & Neurobiology, Boston University, Boston, MA USA; 7 Department of Pediatrics, University of North Carolina at Chapel Hill, Chapel Hill, NC USA; 8 Department of Environmental Sciences and Engineering, University of North Carolina at Chapel Hill, Chapel Hill, NC USA; 9 Department of Genetics, University of North Carolina at Chapel Hill, Chapel Hill, NC USA; 10 Department of Computer Science, University of North Carolina at Chapel Hill, Chapel Hill, NC USA; 11 School of Nursing, University of North Carolina at Chapel Hill, Chapel Hill, NC USA

**Keywords:** Cognitive impairment, Neurodevelopment, Preterm children, Genome-wide association study (GWAS), Latent profile analysis (LPA), Genetic mechanisms

## Abstract

**Background:**

Although survival rates for infants born extremely preterm (gestation < 28 weeks) have improved significantly in recent decades, neurodevelopmental impairment remains a major concern. Children born extremely preterm remain at high risk for cognitive impairment from early childhood to adulthood. However, there is limited evidence on genetic factors associated with cognitive impairment in this population.

**Methods:**

First, we used a latent profile analysis (LPA) approach to characterize neurocognitive function at age 10 for children born extremely preterm. Children were classified into two groups: (1) no or low cognitive impairment, and (2) moderate-to-severe cognitive impairment. Second, we performed TOPMed-based genotype imputation on samples with genotype array data (*n* = 528). Third, we then conducted a genome-wide association study (GWAS) for LPA-inferred cognitive impairment. Finally, computational analysis was conducted to explore potential mechanisms underlying the variant x LPA association.

**Results:**

We identified two loci reaching genome-wide significance (*p* value < 5e-8): TEA domain transcription factor 4 (*TEAD4* at rs11829294, *p* value = 2.40e-8) and syntaxin 18 (*STX18* at rs79453226, *p* value = 1.91e-8). Integrative analysis with brain expression quantitative trait loci (eQTL), chromatin conformation, and epigenomic annotations suggests tetraspanin 9 (*TSPAN9*) and protein arginine methyltransferase 8 (*PRMT8*) as potential functional genes underlying the GWAS signal at the *TEAD4* locus.

**Conclusions:**

We conducted a novel computational analysis by utilizing an LPA-inferred phenotype with genetics data for the first time. This study suggests that rs11829294 and its LD buddies have potential regulatory roles on genes that could impact neurocognitive impairment for extreme preterm born children.

**Supplementary Information:**

The online version contains supplementary material available at 10.1186/s11689-022-09429-x.

## Background

Extreme prematurity (birth < 28 weeks of gestation) remains one of the leading causes of neonatal morbidity and mortality in the USA [1]. Although survival rates for infants born extremely preterm have improved dramatically in recent decades, children born extremely preterm remain at higher risk for cognitive impairment, with lower average general intelligence and executive function deficit [2–6] and 9-fold higher risk of severe cognitive impairment compared to children born full-term [7–13]. Adverse neurodevelopmental outcomes, such as cognitive impairment, affect ~ 1 million preterm infants born each year [14] and may persist through adulthood [15–18]. Although cognitive impairment is not always severe, even mild deficits can have substantial impact, resulting in a spectrum of outcomes from difficulties in school to inability to lead an independent adult life [19]. Specific problems can include deficits in executive function, language, learning and memory, attention, perceptual-motor function, and social cognition [3, 6, 20, 21], which negatively affect well-being [19]. Cognitive impairment has life-long effects on quality of life, with significant familial and social capital costs. Although precise data are limited [22], lifetime costs collectively for children born in 2000 with intellectual disability alone are estimated at $51.2 billion [23].

Despite substantial research efforts to understand neurodevelopment outcomes, we know remarkably little about genetic factors and molecular mechanisms influencing cognitive function in preterm children. Previous genetic studies have discovered hundreds of genetic variants that can predispose children to neurodevelopmental disorders including autism spectrum disorder [24], attention deficit disorder with hyperactivity [25], intellectual disability [26, 27], specific language impairment [28], specific learning disorders [29], and childhood onset schizophrenia [30]. Some genetic studies have evaluated genetic risk factors for neurodevelopmental outcomes for preterm children or children with low birth weight [31–36]. *MAOA* was found to be associated with mental development throughout early childhood among preterm children [31]. A variant rs4074134 of *BDNF*, and a rare insertion/deletion in the intron region of *SLC6A4* were significant predictors of cognitive performance at school age in a study of genetic risk factors for poor cognitive development in children with low birth weight [36]. With preterm infants from a randomized controlled trial (RCT) examining antenatal exposure to corticosteroids, Clark et al. found variants of *IL1B*, *IL4R*, and *IL6* associated with lower scores on the Bayley’s Scales of Infant Development and developmental delay at age 2 [34], and Costantine et al. [33] found that variants of *VIP* and *GRIN3A* were associated with cerebral palsy. A *COMT* variant was associated with reduced corpus callosum size in adults with history of preterm birth [32].

However, previous studies do not explain the pathways through which these variants or genes might influence the risk of poor cognitive outcomes, and few genome-wide association studies (GWAS) examined the genomic regions associated with cognitive function among children born extremely preterm. Therefore, identifying genetic factors that are associated with children’s cognitive function and understanding related mechanisms are necessary to develop earlier screening assessments and effective precision interventions and understand why some preterm children of the same gestational age do worse than others. To advance along these directions, we utilized samples from the extremely low gestational age newborns (ELGAN) cohort [37], the largest US-based study of children born extremely preterm, to identify genetic factors associated with cognitive impairment at age 10 years. Finally, integrative analysis with brain expression quantitative trait loci (eQTL) and chromatin interactome data was performed to identify potential causal variants and functional genes underlying the GWAS associations.

## Methods

### Study participants

ELGAN is a multicenter cohort study originally designed to identify exposures increasing risk of structural and functional neurologic disorders in children born extremely preterm [37]. A total of 1506 infants born before the 28th week of gestation and 1249 mothers were enrolled during the years 2002–2004. Study participants were enrolled at 14 hospitals in the United States to achieve a large enough sample size and generalizability. The enrollment and consent procedures were approved by the individual institutional review boards. At the age of 10 years, 889 of the surviving children returned for follow up (ELGAN2, 92% of the 966 who were recruited for this phase of the ELGAN Study) and were assessed for cognition capacity, learning abilities, and impairments in executive function [7]. Of these children, 528 had genotype data available for analysis and thus constitute the sample size of this paper. Table 1 summarizes demographic information for the ELGAN2 cohort and the ELGAN2 subset with genetic data (*n* = 528) we used in our analysis.

**Table 1:** Participant characteristics of the ELGAN2 subset and ELGAN2 cohort

Variable name	ELGAN2 subset (*N* = 528)	ELGAN2 (*N* = 889)
	*n* (% or SD)	*n* (% or SD)
Infant sex		
Male	274 (51.9%)	455 (51.2%)
Female	254 (48.1%)	434 (48.8%)
Cognitive impairment		
No/Low	390 (73.9%)	660 (74.2%)
Moderate/Severe	138 (26.1%)	214 (24.1%)
Not reported	0	15 (1.7%)
Gestational age	26.1 (1.27)	26.1 (1.28)
Maternal education		
≤ 12 years	205 (38.8%)	355 (39.9%)
13–15 years	119 (22.5%)	202 (22.7%)
16+ years	204 (38.6%)	306 (34.4%)
Not reported	0	26 (2.9%)
Maternal smoking		
Yes	128 (24.2%)	215 (24.2%)
No	400 (75.8%)	655 (73.7%)
Not reported	0	19 (2.1%)
Race		
White	342 (64.8%)	554 (62.3%)
Black	133 (25.2%)	227 (25.5%)
Other	53 (10.0%)	98 (11.0%)
Not reported	0	10 (1.1%)
Public insurance		
Yes	167 (31.6%)	307 (34.5%)
No	361 (68.4%)	568 (63.9%)
Not reported	0	14 (1.6%)
Multiple births		
Yes	189 (35.8%)	313 (35.2%)
No	339 (64.2%)	576 (64.8%)

### Cognitive function at age 10 years

Cognitive function at age 10 years was assessed with latent profile analysis (LPA) [38], which empirically identifies subgroups of children who share similar profiles on a set of measures. The LPA included 9 cognitive measures including verbal and nonverbal intelligence quotient (IQ) and several measures of executive function (EF). IQ was assessed with the School-Age Differential Ability Scales–II (DAS-II) Verbal and Nonverbal Reasoning scales. EF was assessed with two subtests from the DAS-II and five subtests from the Developmental NEuroPSYchological Assessment-II (NEPSY-II). Working memory was evaluated with the DAS-II Recall of Digits Backwards and Recall Sequential Order test. The NEPSY-II Auditory Attention and Auditory Response Set, Animal Sorting Inhibition, and Inhibition Switching subtests were utilized to examine auditory attention and set switching, concept generation and mental flexibility, and simple inhibition and inhibition shifting, respectively [7]. It has been shown that characterizing cognitive function using measures of executive function in addition to IQ better discriminates the academic performance and educational needs of children born extremely preterm [38]. LPA classifies subjects who share a similar pattern of scores on the measured variables, while maximizing the difference in scoring patterns across distinct profiles [39]. It assigns subjects into a finite number of profiles by identifying the most likely model that describes the heterogeneity of data, which is known as finite mixture models.

To determine the optimal number of profiles, LPA was fit to the data, and Bayesian information criteria (BIC) [40], sample-size-adjusted Bayesian information criteria (SSABIC) [41], and Lo–Mendell–Rubin-adjusted (LMR) likelihood ratio test [42] were used to assess model fit. Children were categorized by their most likely latent profile for further analysis. In this sample, a four-profile model provided the best fit for the data [38]. For our analysis, we used a binary classification that grouped participants into two previously validated distinct profile groups (LPAx) [38, 43]: no or low cognitive impairment and moderate-to-severe cognitive impairment.

### Genotype data

Genomic DNA was isolated from umbilical cords and genotyping was performed using Illumina 1 Million Quad (Illumina Inc, San Diego, California). This work was done as part of the candidate gene analysis of severe intraventricular hemorrhage (IVH) in preterm born infants [44], where infants with birth weights 500–1250 g and severe grades IVH and neonates with normal cranial ultrasounds were enrolled prospectively at 24 universities. A subset of ELGAN participants were provided as additional samples along with samples from a few other studies in the IVH study [44].

We performed variant level and sample level quality control (QC) on genotype data. For variant level QC, we excluded variants with call rate < 90% or minor allele frequency (MAF) < 1%. For sample level QC, we excluded samples with missing rate > 10%. These resulted in 700,845 SNPs and 528 samples using plink v.1.90 [45, 46].

### Genotype imputation

Starting with the quality controlled (QCed) genotype data, we used the Michigan imputation server [47] for phasing and imputation using TOPMed freeze 5 [48] as the reference panel. Specifically, Eagle [49] was used for phasing and Minimac4 [50, 51] was used for imputation. We performed strand matching by dropping ambiguous (i.e., A/T or C/G) SNPs and by flipping non-ambiguous SNPs that were initially in − strand when compared to alleles in the + strand observed in the TOPMed freeze 5 reference panel. Genotype data was lifted over to genome build hg38. In total, we obtained ~ 34 million well imputed variants, with 5.5 million variants having MAF > 5%, 10.5 million variants having MAF > 1%, and 17.4 million variants having MAF > 0.5%. Well imputed variants were defined by having Rsq > 0.8, where Rsq is the estimated imputation quality metric [50, 51]. To evaluate the imputation accuracy, we randomly selected 5% of the genotyped variants on chromosome 1 and performed genotype imputation with the rest 95% genotyped variants, again with the TOPMed freeze 5 reference panel. The 5% masked genotyped variants were saved for imputation quality evaluation. Specifically, we calculated the squared Pearson correlation between imputed genotypes and true observed genotypes. For the 1956 variants on chromosome 1 tested, the mean squared Pearson correlation was 0.97, which suggests that most variants were well imputed even with a relatively small sample size (Fig. S1, Additional file 1), consistent with what has been reported in the literature [52–54].

### Genome-wide association analysis

We used EPACTS 3.3.0 [55] for single variant association testing. To account for relatedness among samples, we used the EMMAX (Efficient Mixed Model Association eXpedited) test [56], which is an efficient implementation of mixed model association accounting for sample structure including population structure and hidden relatedness. Biallelic SNPs with MAF > 2% (did not account for relatedness) and Rsq > 0.8 were included in the analysis. In total, 8,535,130 variants were included in the association analysis. For the 528 samples who had genotype and covariates data available, we inferred kinship matrix using EPACTS and top 10 principal components (PCs) from the genotype data using PLINK. We performed the association test on the outcome LPAx, a binary outcome that classifies children into no or low cognitive impairment and moderate-severe cognitive impairment groups. The covariates for the single variant association analysis included gestational age, maternal education, maternal race, sex of the infant, and top 10 PCs. We performed sensitivity analysis for variants showing suggestive signals by further adjusting interaction terms between covariates.

## Results

### Association analysis results

**Fig. 1:**
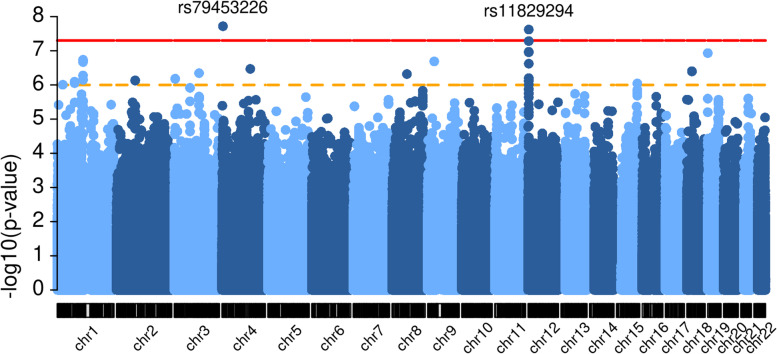
Manhattan plot. The Manhattan plot visualizes the association of SNPs along the genome with the LPAx trait. *X*-axis represents genomic location and y-axis represents -log10(*p* value). Each dot represents a SNP tested. SNPs above the red horizontal line, which marks the 5 × 10^−8^ are considered genome-wide significant. This plot was generated using the R package *karyoploteR* [57]. NCBI build 38

**Fig. 2:**
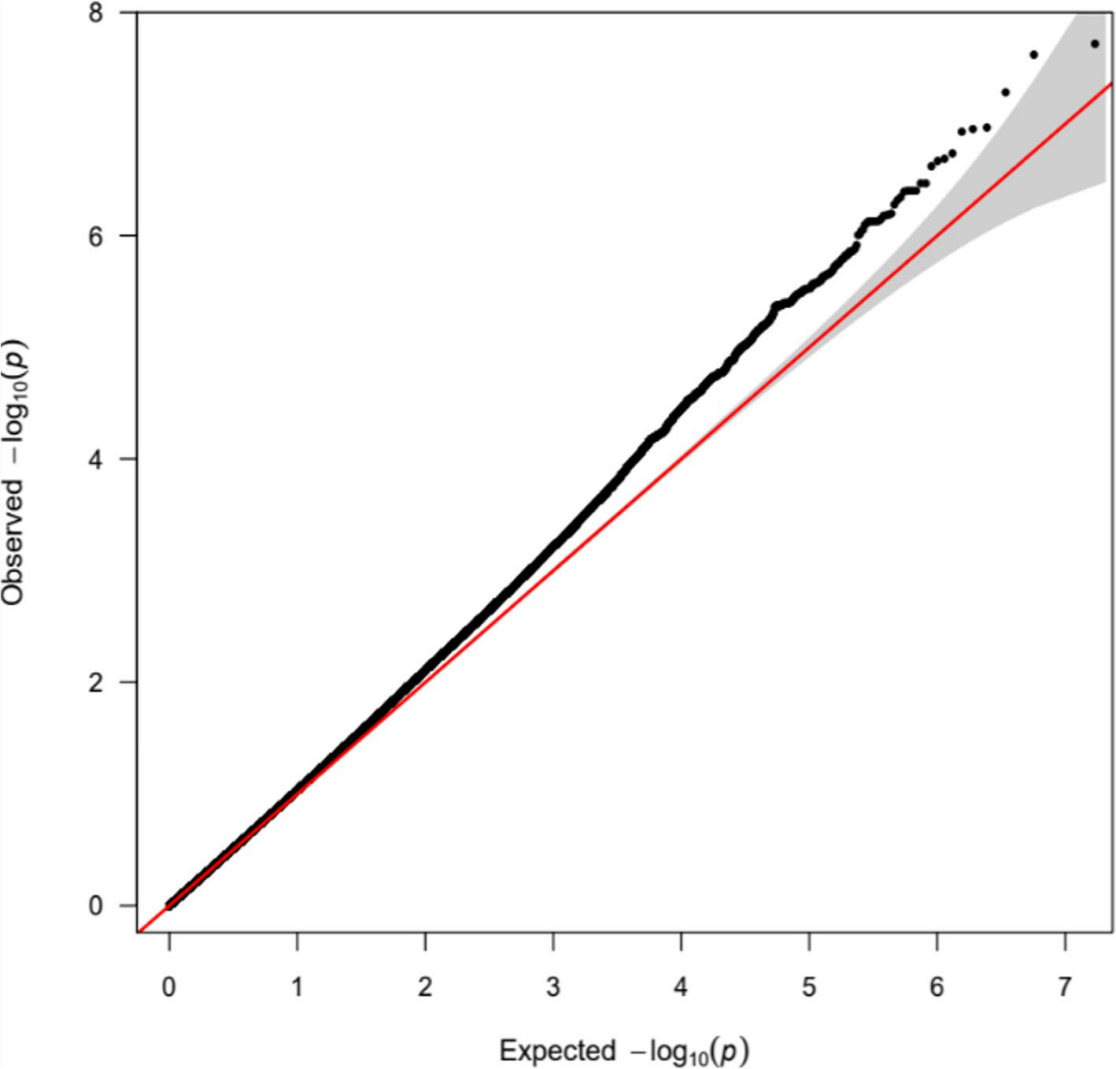
QQ plot. A quantile-quantile (Q-Q) plot is used to characterize the extent to which the observed distribution of the test statistics follows the expected null distribution. This plot was generated using the R package *qqman* [58]

We conducted a GWAS on LPAx of 528 samples from the ELGAN2 cohort. We identified two genome-wide significant loci from the 8,535,130 variants tested: *STX18* and *TEAD4*, which are located on chromosome 4 and chromosome 12, respectively (Fig. 1). The index SNPs are rs79453226 (MAF = 0.036) and rs11829294 (MAF = 0.145) at the *STX18* and *TEAD4* loci, respectively. The genomic inflation factor λ, which measures the inflation in the test statistics and is calculated as the ratio of the median of the empirically observed distribution of the test statistics to the expected median (median of a chi-square distribution with one degree of freedom), is 1.038, which suggests no significant inflation of test statistics or excess false positive rate (Fig. 2). Table 2 shows genome-wide significant variants, and suggestive variants with *p* values less than 1e-6. For this set of suggestive variants, we performed additional association analysis by including interaction terms between non-genetic covariates (gestational age, maternal education, maternal race, and sex of the infant). We evaluated each interaction term separately, and we found that the two genome-wide significant loci remained significant and most suggestive loci had similar significance levels as in the original model (Fig. S2, Additional file 1), indicating that our top loci are robust to the further adjustment of interactions between covariates.

**Table 2:** Significant and suggestive association results for LPAx

rsID	Chr^a^	Position^a^	REF	ALT	*P* value	MAF	Locus	Effect size (s.e.)
rs79453226	chr4	4483114	G	C	1.91e-08	0.036	*STX18* (intron)	0.421 (0.074)
rs11829294	chr12	3014153	C	T	2.40e-08	0.145	*TEAD4* (intron)	− 0.231 (0.041)
rs10774094	chr12	3014630	C	A	5.21e-08	0.160	*TEAD4* (intron)	− 0.214 (0.039)
rs12322215	chr12	3001421	G	T	1.08e-07	0.142	*TEAD4* (intron)	− 0.215 (0.040)
rs10128796	chr12	3003552	G	A	1.11e-07	0.142	*TEAD4 (intron)*	− 0.214 (0.040)
rs73916918	chr19	376264	C	T	1.17e-07	0.020	*THEG* (5’ UTR)	0.522 (0.097)
rs59359613	chr1	113154555	C	T	1.83e-07	0.023	intergenic	0.465 (0.088)
rs16913588	chr9	28733517	T	C	2.05e-07	0.036	intergenic	0.396 (0.075)
rs58545250	chr1	113172866	T	C	2.14e-07	0.024	*RP11-389O22.4* (downstream)	0.438 (0.083)
rs61114884	chr12	3004684	T	A	2.39e-07	0.135	*TEAD4* (intron)	− 0.210 (0.040)
rs28411755	chr4	124309484	T	C	3.40e-07	0.025	intergenic	0.431 (0.083)
rs7657348	chr4	124310584	A	G	3.40e-07	0.025	intergenic	0.431 (0.083)
rs76946462	chr18	22326760	A	G	3.96e-07	0.057	intergenic	0.278 (0.054)
rs77039990	chr18	22327483	G	A	3.97e-07	0.057	intergenic	0.278 (0.054)
rs76500624	chr18	22326662	G	A	3.97e-07	0.057	intergenic	0.278 (0.054)
rs75050632	chr18	22327123	G	A	4.03e-07	0.057	intergenic	0.277 (0.054)
rs115606157	chr3	108839197	T	G	4.53e-07	0.030	*TRAT1* (intron)	0.385 (0.07)
rs73690518	chr8	65242418	C	T	4.80e-07	0.048	intergenic	0.355 (0.070)
rs17031018	chr1	113100296	A	G	5.27e-07	0.035	*LRIG2* (intron)	0.377 (0.074)
rs61917974	chr12	3011978	T	C	6.33e-07	0.109	*TEAD4* (intron)	− 0.223 (0.044)
rs12296242	chr12	3006641	G	C	6.49e-07	0.133	*TEAD4* (intron)	− 0.202 (0.040)
rs143601180	chr3	4370781	A	G	6.56e-07	0.032	*SUMF1* (intron)	0.383 (0.076)
rs79946490	chr3	4385952	C	T	6.64e-07	0.032	*SUMF1* (intron)	0.383 (0.076)
rs17031120	chr1	113144809	T	C	7.12e-07	0.021	intergenic	0.462 (0.092)
rs2163633	chr2	81884390	C	A	7.36e-07	0.046	intergenic	− 0.350 (0.070)
rs6716465	chr2	81871292	G	C	7.43e-07	0.045	intergenic	− 0.350 (0.070)
rs11062457	chr12	3010236	C	T	7.44e-07	0.145	*TEAD4* (intron)	− 0.201 (0.040)
rs72921448	chr2	81824332	T	C	7.44e-07	0.045	intergenic	− 0.350 (0.070)
rs2286647	chr12	3010912	C	T	7.46e-07	0.145	*TEAD4* (intron)	− 0.201 (0.040)
rs116629423	chr2	81858789	A	G	7.47e-07	0.045	intergenic	− 0.350 (0.070)
rs143923810	chr12	2988024	C	T	7.73e-07	0.139	*TEAD4* (intron)	− 0.194 (0.039)
rs10493588	chr1	76227682	C	T	8.04e-07	0.057	*ST6GALNAC3* (intron)	0.279 (0.056)
rs17098434	chr1	76232427	G	A	8.85e-07	0.057	*ST6GALNAC3* (intron)	0.277 (0.056)
rs8025099	chr15	91488748	C	A	9.06e-07	0.486	*CRAT37* (intron)	− 0.129 (0.026)
rs12318430	chr12	3006040	C	A	9.75e-07	0.132	*TEAD4* (intron)	− 0.201 (0.040)
rs9424366	chr1	24475103	G	C	9.86e-07	0.036	*NIPAL3* (downstream)	0.349 (0.070)

Ordered by significance

^a^NCBI build 38

**Fig. 3:**
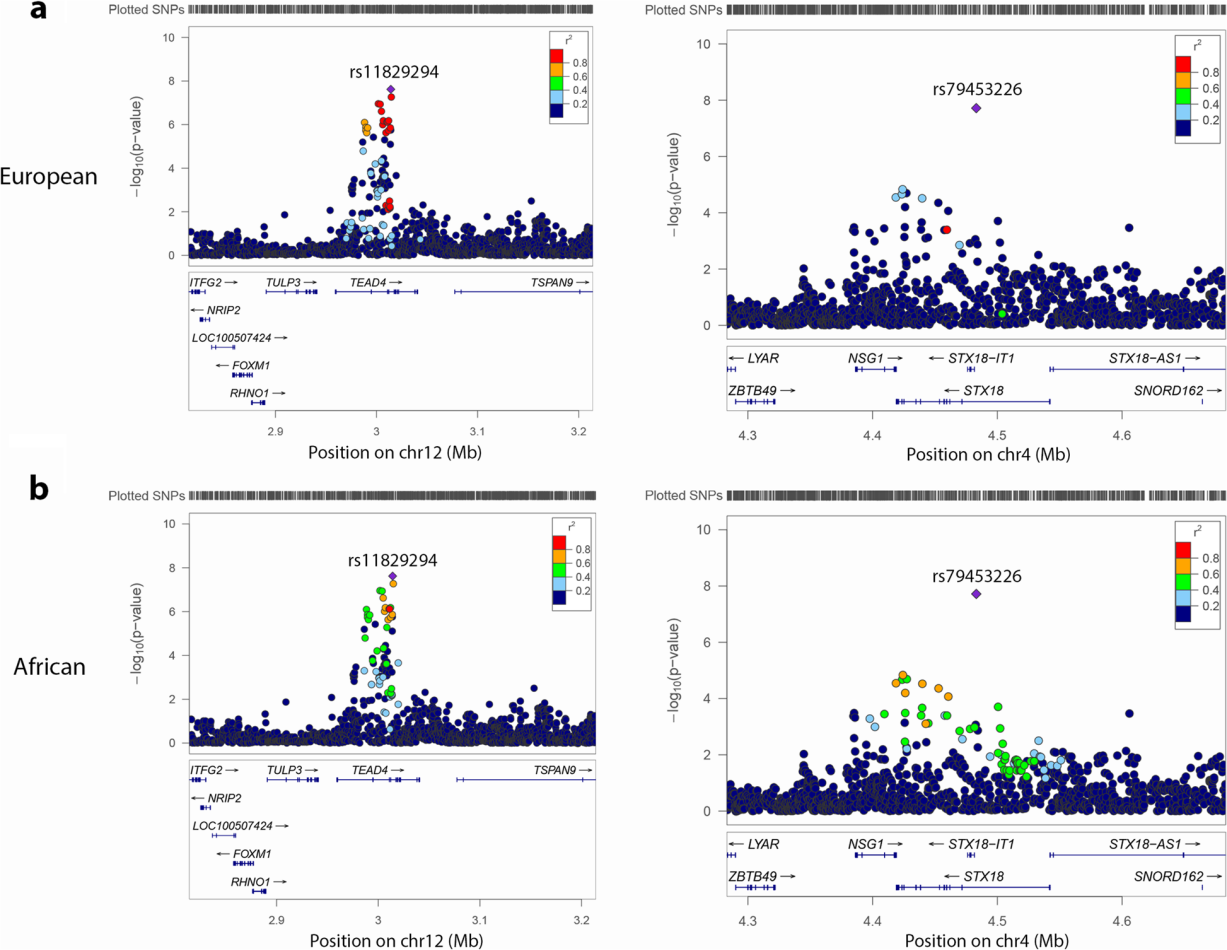
Locus zoom plots for the two genome-wide significant loci. Colors represent linkage disequilibrium r^2^ values calculated from TOPMed individuals with the lead SNP in each plot. **a** Locus zoom plots with linkage disequilibrium r^2^ values calculated from TOPMed European ancestry individuals. **b** Locus zoom plots with linkage disequilibrium *r*^2^ values calculated from TOPMed African ancestry individuals. NCBI build 38

Figure 3 shows LocusZoom [59] plots for the two genome-wide significant loci, with linkage disequilibrium (LD) from TOP-LD [60], calculated using TOPMed European and African participants. We can see that in the European population, the lead variant rs11829294 in the *TEAD4* region has a number of LD tags (e.g., 21 variants with *r*^2^ ≥ 0.8) and some of them had highly significant *p* values; by contrast, the lead variant rs79453226 in the *STX18* region has fewer LD tags (2 variants with *r*^2^ ≥ 0.8) that showed suggestive association (Fig. 3a). In the African population, the lead variant rs11829294 in the *TEAD4* region has only 2 LD tags, which did not have significant or suggestive association, and the lead variant rs79453226 in the *STX18* region has no LD tag with *r*^2^ ≥ 0.8 (Fig. 3b).

### Epigenetic functional annotations

To further investigate the two loci identified for potential mechanisms, we examined several functional annotation metrics, including the CADD phred score [61] and the fathmm MKL score [62]. CADD phred score measures the deleteriousness of variants and is computed as -10*log10(rank/total). A CADD phred score of ≥ 10 indicates that the variant is predicted to be among the 10% most deleterious variants in the human genome, a score of ≥ 20 indicates among the 1% most deleterious. The fathmm MKL score predicts the functional consequences of variants where values above 0.5 are generally considered deleterious, and values below 0.5 neutral or benign. We also looked at the Genehancer feature [63] and the genes predicted by Genehancer. Table 3 shows functional annotations for variants that passed the suggestive *p* value threshold (*p* value < 1e-6). We observed that variants rs9424366, rs79946490, rs58545250, and rs17031018 were among the top 10% most deleterious in the human genome, and variant rs16913588 was predicted to be deleterious (with a fathmm MKL score of 0.97). Several variants were assigned by Genehancer as falling into enhancer regions with target genes*TSPAN9*, *ITPR1*, and *CLIC4*. These results provide evidence that some of the variants might have deleterious effects that are relevant to neurocognitive development in preterm children and suggest additional genes that might be functionally related.

**Table 3:** Epigenetic functional annotations for selected genome-wide significant and suggestive associations

rsID	***P*** value	CADD phred	FathmmMKL	Genehancer feature	Genehancer connected gene	Locus
rs11829294	2.40e-08	3.728	0.21	enhancer	*TSPAN9*	*TEAD4* (intron)
rs10774094	5.21e-08	0.805	0.10	enhancer	*TSPAN9*	*TEAD4* (intron)
rs16913588	2.05e-07	7.525	0.97	–	–	intergenic
rs58545250	2.14e-07	9.661	0.49	–	–	*RP11-389O22.4* (downstream)
rs61114884	2.39e-07	3.602	0.15	enhancer	*TSPAN9*	*TEAD4* (intron)
rs17031018	5.27e-07	9.16	0.30	–	–	*LRIG2* (intron)
rs79946490	6.64e-07	10.19	0.23	enhancer	*ITPR1*	*SUMF1* (intron)
rs11062457	7.44e-07	0.362	0.13	enhancer	*TSPAN9*	*TEAD4* (intron)
rs2286647	7.46e-07	0.16	0.07	enhancer	*TSPAN9*	*TEAD4* (intron)
rs143923810	7.73e-07	1.518	0.04	enhancer	*TSPAN9*	*TEAD4* (intron)
rs9424366	9.86e-07	13.82	0.13	enhancer	*CLIC4*	*NIPAL3* (downstream)

### Chromatin interactions

**Fig. 4:**
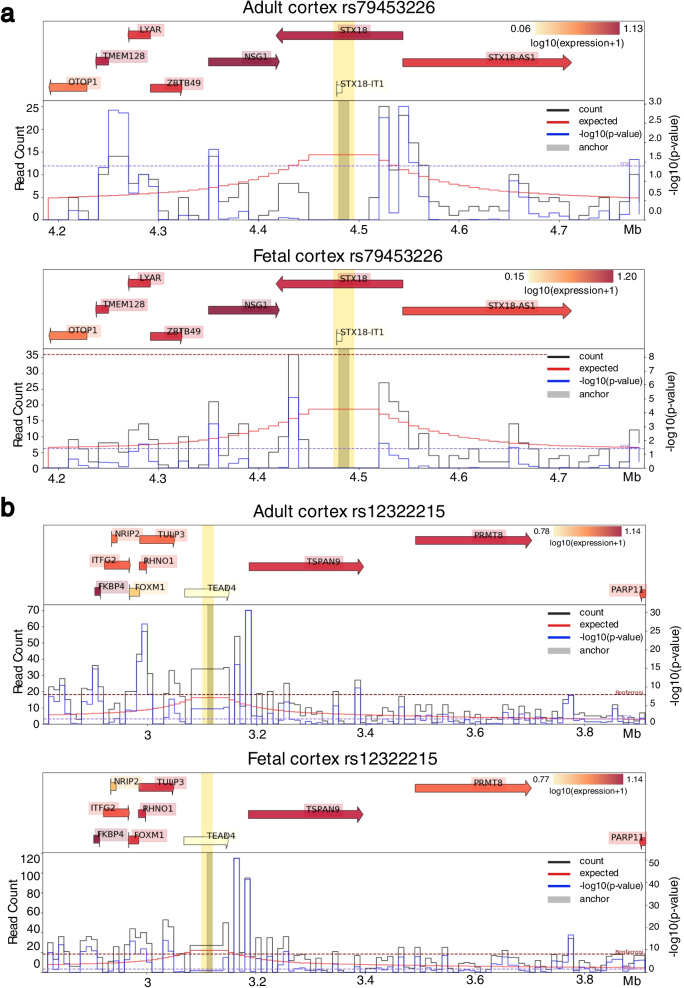
Virtual 4C plots. Centered at **a** rs79453226 **b** rs12322215 in adult cortex and fetal cortex. The bin containing the anchor position is indicated as a thick grey vertical bar. Different genes or regions can be highlighted in yellow. On the top is gene expression data with gene locations. Each gene is indicated by an arrow pointing the direction of transcription. The start site is indicated by the tail of the arrow. Each gene is labeled by its common name and highlighted in red indicating the expression level: the deeper the red color the higher the expression. On the bottom is the chromatin interaction Hi-C data that is plotted as a virtual 4C plot with the given anchor position. The black line shows the observed counts, the red line shows the expected counts, and the blue line shows the -log10(*p* value). The range of the -log10(*p* value) is plotted on the *y*-axis on the right while the range of the count data is shown on the left. The *x*-axis is the genomic location in Mb. NCBI build 37

We examined chromatin conformation data for additional functional implications based on physical contacts from Hi-C and alike technologies. Figure 4 shows virtual 4C plots generated by HUGIn2 [64] for the top two loci in adult cortex and fetal cortex Hi-C data [65]. HUGIn2 is a web-based viewer of genome-wide chromatin conformation data to explore chromatin spatial organization across multiple human cell lines and primary tissues. HUGIn2 can additionally incorporate data from multiple sources including genetic variants, chromatin organization features (e.g., topologically associating domains (TADs) [66], frequently interacting regions (FIREs) [67]), gene expression, and epigenetic annotations. For our purpose, we examined ± 500 kb regions around each locus. Significant chromatin interactions between the putative regulatory regions (harboring some GWAS variant(s)) and promoters of genes suggest the likely causal or effector genes regulated by the GWAS variant(s). The results were consistent between adult cortex and fetal cortex. The variant rs79453226 at the *STX18* locus was linked to the promoter regions of several genes, including *STX18* and *NSG1* (Fig. 4a), and the variant rs12322215 at the *TEAD4* locus was linked to *FKBP4*, *FOXM1*, *RHNO1*, *TULP3*, *TSPAN9*, and *PRMT8* (Fig. 4b).

### Overlapping with brain eQTL

Next, we investigated whether we could find any brain eQTL signals among the top variants. We examined all variants with LD *r*^2^ ≥ 0.6 with variants that passed the suggestive *p* value threshold (*p* value < 1e-6) using LD calculated from TOPMed European ancestry samples. Table 4 shows variants overlapped with commonMind eQTL [68] with FDR < 5%. Multiple brain eQTLs for *PRMT8* on chromosome 12 in LD with the index SNP rs11829294 were identified.

**Table 4:** Variants overlapped with commonMind eQTL

rsID	Gene	Chr^**a**^	Position^**a**^	FDR	Index SNP	LD r^**2**^ with the index SNP
rs143923810	*PRMT8*	chr12	2988024	0.010	rs11829294	0.724
rs7302783	*PRMT8*	chr12	2989245	0.010	rs11829294	0.724
rs7302789	*PRMT8*	chr12	2989254	0.010	rs11829294	0.720
rs10082968	*PRMT8*	chr12	2990125	0.025	rs11829294	0.720
rs12322215	*PRMT8*	chr12	3001421	0.048	rs11829294	0.883
rs10128796	*PRMT8*	chr12	3003552	0.045	rs11829294	0.883

^a^NCBI build 38

### Overlapping with selective sweeps

We also examined whether the identified top variants overlapped with selective sweeps detected by S/HIC [69]. Table 5 shows multiple variants in LD (*r*^2^ ≥ 0.6) with the top variants overlapped with selective sweeps: all except one on chromosome 12 at the *TEAD4* locus and one in an intergenic locus on chromosome 2. We found that some variants at the *TEAD4* locus were located in soft sweep regions, where selection on standing variation produced qualitatively different skews in LD and allele frequencies.

**Table 5:** Variants overlapped with selective sweeps

Chr^a^	Position^a^	Start	End	Selective sweeps
chr12	3095812	2800000	3100000	CEU: soft, GWD: soft, LWK: soft, PEL: soft, YRI: soft
chr12	3097190	2800000	3100000	CEU: soft, GWD: soft, LWK: soft, PEL: soft, YRI: soft
chr12	3098411	2800000	3100000	CEU: soft, GWD: soft, LWK: soft, PEL: soft, YRI: soft
chr12	3098420	2800000	3100000	CEU: soft, GWD: soft, LWK: soft, PEL: soft, YRI: soft
chr12	3099291	2800000	3100000	CEU: soft, GWD: soft, LWK: soft, PEL: soft, YRI: soft
chr2	82111514	82100000	82200000	GWD: soft, YRI: soft

Population: CEU (UT, USA), GWD (Western Divisions, the Gambia), LWK (Webuye, Kenya), PEL (Lima, Peru), YRI (Ibadan, Nigeria). Start and end are start and end positions of selective sweep regions

^a^NCBI build 37

## Discussion

Cognitive impairment is highly prevalent among children born extremely preterm. Yet, limited evidence is available on the genetic factors that may contribute to this kind of impairment. In this study, we aimed to identify genetic factors that are associated with children’s cognitive function and understand related genetic mechanisms by utilizing samples from the ELGAN cohort. Leveraging an LPA-derived phenotype and genetics data, we identified two genome-wide significant loci in our genome-wide association analysis for LPAx (a data-derived cognitive impairment outcome): *TEAD4* (rs11829294, *p* value = 2.40e-8) and *STX18* (rs79453226, *p* value = 1.91e-8).

We utilized chromatin conformation data from multiple human cell lines and primary tissues to see whether there are significant chromatin interactions between the two genome-wide significant loci and their neighboring regions. In adult cortex and fetal cortex, we found that variant rs12322215 (*p* value = 1.08e-07) in high LD with rs11829294 (*r*^2^ = 0.883) is linked to promoter regions of a few genes including *TSPAN9* and *PRMT8* (Fig. 4). Furthermore, the association at the *TEAD4* locus rs11829294 and a few other variants that showed suggestive significance at the same locus were assigned by Genehancer as falling into the enhancer region of *TSPAN9* (Table 3). We also observed *TSPAN9* is highly expressed in both adult cortex and fetal cortex but not in hippocampus, and we did not observe similar chromatin interactions in hippocampus (Fig. 4). *TSPAN9* is located at chr12:3,077,355-3,286,564 (GRCh38/hg38) and is one of tetraspanins, a superfamily of glycoproteins that function as “organizers” of cell membranes by recruiting other receptors and signaling proteins into tetraspanin-enriched microdomains and induce normal platelet activation [70]. Those proteins mediate signal transduction events that play a role in the regulation of cell development, activation, growth, and motility. *TSPAN9* is highly expressed in normal brain tissues, including cerebellum and cerebellar hemisphere [71]. These pieces of evidence suggest the potential regulatory role of rs11829294 and its LD buddies on the *TSPAN9* gene that could impact cognitive development among children born extremely preterm*.*

We also performed integrative analysis with brain eQTL to identify potential functional genes underlying the genome-wide significant association. A few brain eQTL for *PRMT8* were found to be in high LD with rs11829294 (Table 4). *PRMT8* is a member of the protein arginine *N*-methyltransferase (PRMT) family, which mediates protein arginine methylation, a common post-translational modification that has been implicated in signal transduction, RNA processing, transcriptional regulation, and DNA repair [72]. *PRMT8* was found to be associated with the plasma membrane and has a tissue-specific expression in brain. Specifically, it is highly expressed in nucleus accumbens (basal ganglia), putamen (basal ganglia), cortex, caudate (basal ganglia), frontal cortex (Brodmann area 9), and anterior cingulate cortex (Brodmann area 24) [71]. It was also identified as a tissue-restricted enzyme responsible for proper asymmetric dimethylarginine (ADMA) level in postmitotic neurons where *PRMT8*-dependent arginine methylation is required for neuroprotection against age-related increased of cellular stress [73]. Moreover, *PRMT8* in human embryonic stem cells (hESCs) plays an important role not only in maintaining pluripotency but also in controlling mesodermal differentiation [74]. Along with the evidence that variant rs12322215 is linked to the promoter region of *PRMT8*, we conclude that *PRMT8* is another biologically plausible gene regulated by eQTL at the *TEAD4* locus that could have potential effects on cognitive impairment among preterm children.

Additionally, we found that multiple variants in LD with rs11829294 overlapped with selective sweeps detected by S/HIC (Table 5). There is evidence that soft sweeps are widespread and account for the vast majority of recent human adaptation, and positive selection may often proceed via “soft sweeps” acting on mutations already present within a population. Furthermore, linked positive selection affects patterns of variation across much of the genome, and may increase the frequencies of deleterious mutations [69]. Therefore, these variants in soft sweep regions provide additional evidence for the associations found at the *TEAD4* locus being biologically plausible and potentially causal.

For rs79453226, we found that it is linked to promoter regions of *STX18* and *NSG1* (Fig. 4). We did not find as much evidence for the *STX18* locus supporting the significant association as for the *TEAD4* locus. By examining LD *r*^2^ values calculated from TOPMed, we observed that rs11829294 and rs79453226 have different LD structures at their loci (Fig. 3). Specifically, rs11829294 has a number of LD buddies with *r*^2^ ≥ 0.8 showing suggestive association with LPAx in the European population. In contrast, rs79453226 has fewer LD buddies and is not in high LD with any of the suggestive variants. Therefore, rs11829294 is more likely to tag effects from causal variants than rs79453226.

With association results and other information considered, we did not have direct evidence indicating *TEAD4* and *STX18* as causal genes. However, there is evidence that *TEAD4* and *STX18* are related to placental development and brain respectively.*TEAD4* is a member of the TEAD transcription factor family, which is best known for transcriptional output of the Hippo signaling pathway and has been implicated in processes such as development, cell growth and proliferation, tissue homeostasis, and regeneration [75]. TEADs have been found to be evolutionarily conserved, and have been shown to play important roles in various biological processes and human disease [76, 77]. Mouse knockout studies showed that *TEAD4* is specifically required for embryo implantation and trophectoderm lineage determination [78, 79], which play important roles in placental development. *TEAD4* null mice are embryonic lethal due to failure in embryo implantation; however, disruption of *TEAD4* after embryo implantation results in normal development [78, 79]. TEADs seem to have important biological functions, but studies thoroughly characterizing TEAD function and regulation are lacking. In the future, we can utilize genome-wide DNA methylation, mRNA, and miRNA data from the placenta to study this gene more closely. The gene *STX18* encodes a member of the syntaxin family of soluble *N*-ethylmaleimide-sensitive factor attachment protein receptors (SNAREs) which is part of a membrane tethering complex that includes other SNAREs and several peripheral membrane proteins, and is involved in vesicular transport between the endoplasmic reticulum (ER) and the Golgi complex [80]. It has also been shown that *STX18* is important for the organization of two ER subdomains, smooth/rough ER membranes and ER exit sites by mediating the fusion of retrograde membrane carriers with the ER membrane [81]. Knockdown of *STX18* caused a global change in ER membrane architecture, leading to the segregation of the smooth and rough ER. Moreover, the organization of ER exit sites was markedly changed concomitantly with dispersion of the ER-Golgi intermediate compartment and the Golgi complex. Variants in *STX18* were previously found to be associated with brain volume measurement and neuroimaging measurement [82, 83].

One limitation of our analysis is that our results may not be generalizable to children who are not extreme premature. Another issue is the small sample size, although we were able to impute most variants well (Fig. S1, Additional file), it limits the statistical power of the association analysis. The few genome-wide significant single variant associations we found, and the non-statistically significant heritability estimate also suggest the need for better powered analyses (Additional file 1). It is also possible that variants included in our analyses are in low or moderate LD with true causal variants which are rare and cannot be well-imputed in the ELGAN2 cohort. While ELGAN2 is the largest cohort with genotype and long-term cognitive assessment for extremely preterm children currently available in the USA, in the future we hope to study a larger population with longitudinal data of cognitive function, to investigate whether there are genetic variants that interact with perinatal and neonatal immune factors to increase risk for development of trajectories of impaired cognitive function.

## Conclusions

In this work, we present an innovative computational approach that combines LPA with multi-faceted genomic analysis to investigate potential genetic risk factors underlying cognitive impairment among children born extremely preterm. Our association analysis identified two genome-wide significant loci: *TEAD4* at rs11829294 and *STX18* at rs79453226. Further genomic analysis suggests that rs11829294 and its LD buddies have potential regulatory roles on likely functionally relevant genes *TSPAN9* and *PMRT8*. This study provides new mechanistic insight into neurocognitive function among children born extremely preterm by performing an imputation-based GWAS with subsequent prioritization of causal variants and effector genes.

## Supplementary Information

**Additional file 1.** SNP-heritability estimation with GCTA, Fig. S1, and Fig. S2.

## Data Availability

The genotype data analyzed during the current study are not publicly available but are available from the corresponding author on reasonable request. Epigenetic functional annotations, chromatin interaction, brain eQTL, and selective sweeps data are publicly available.

## Abbreviations

LPA: Latent profile analysis
GWAS: Genome-wide association study
eQTL: Expression quantitative trait loci
RCT: Randomized controlled trial
ELGAN: Extremely low gestational age newborns
IQ: Intelligence quotient
EF: Executive functioning
DAS-II: Differential Ability Scales–II
NEPSY-II: NEuroPSYchological Assessment-II
BIC: Bayesian information criteria
SSABIC: Sample-size-adjusted Bayesian information criteria
LMR: Lo–Mendell–Rubin
IVH: Intraventricular hemorrhage
QC: Quality control
MAF: Minor allele frequency
PCs: Principal components
GRM: Genetic relationship matrix
REML: Restricted maximum likelihood
Q-Q: Quantile-quantile
LD: Linkage disequilibrium
FIREs: Frequently interacting regions
ADMA: Asymmetric dimethylarginine
hESCs: Human embryonic stem cells
SNAREs: Soluble *N*-ethylmaleimide-sensitive factor attachment protein receptors
ER: Endoplasmic reticulum
