# Supplementary material for: Innovative computational approaches shed light on genetic mechanisms underlying cognitive impairment among children born extremely preterm
Source: J Neurodev Disord. 2022 Mar 3;14:16. doi: 10.1186/s11689-022-09429-x (PMC8903548; doi:10.1186/s11689-022-09429-x)
Supplement: Supplementary file 1 — Additional file 1. SNP-heritability estimation with GCTA, Fig. S1, and Fig. S2. [file 11689_2022_9429_MOESM1_ESM.docx]

**SNP-heritability estimation with GCTA**

GCTA [1,2] was used to estimate SNP-heritability for LPAx. We used well imputed SNPs (Rsq>0.8) with MAF > 1%. Since there were many closely related individuals in our sample, we utilized a method that can estimate pedigree-based and SNP-based heritability simultaneously in one model [2]. The main advantage of this method is that it allows us to estimate SNP-based heritability without having to remove related individuals. A genetic relationship matrix (GRM) was first derived using a total of 9,817,454 variants for 22 autosomes using all 528 samples. Another GRM was then made setting the first GRM off-diagonal elements that were below 0.05 to 0. Heritability was estimated by REML (restricted maximum likelihood) analysis with these two GRMs adjusting for covariates (sex, race, maternal education, gestational age) and first 10 principal components. Using all 528 genotyped samples, the estimated SNP-heritability of LPAx is 0.38 (s.e.=1.38) with a prespecified prevalence of 25%. The point estimates of SNP-heritability were moderate but were not significantly different from zero, likely due to our relatively small sample size with closely related individuals.


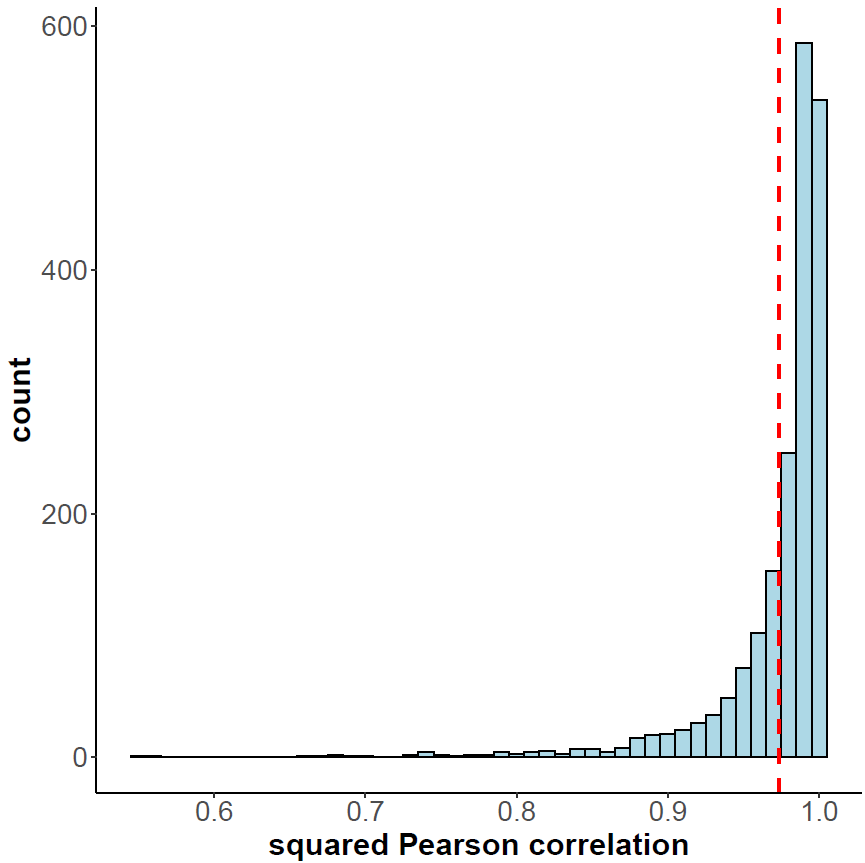


**Fig. S1 Histogram of imputation accuracy on chromosome 1.** The histogram shows squared Pearson correlation between imputed genotypes and observed genotypes for 1,956 variants randomly masked on chromosome 1 when performing imputation. The red dashed line represents the average squared Pearson correlation.

**
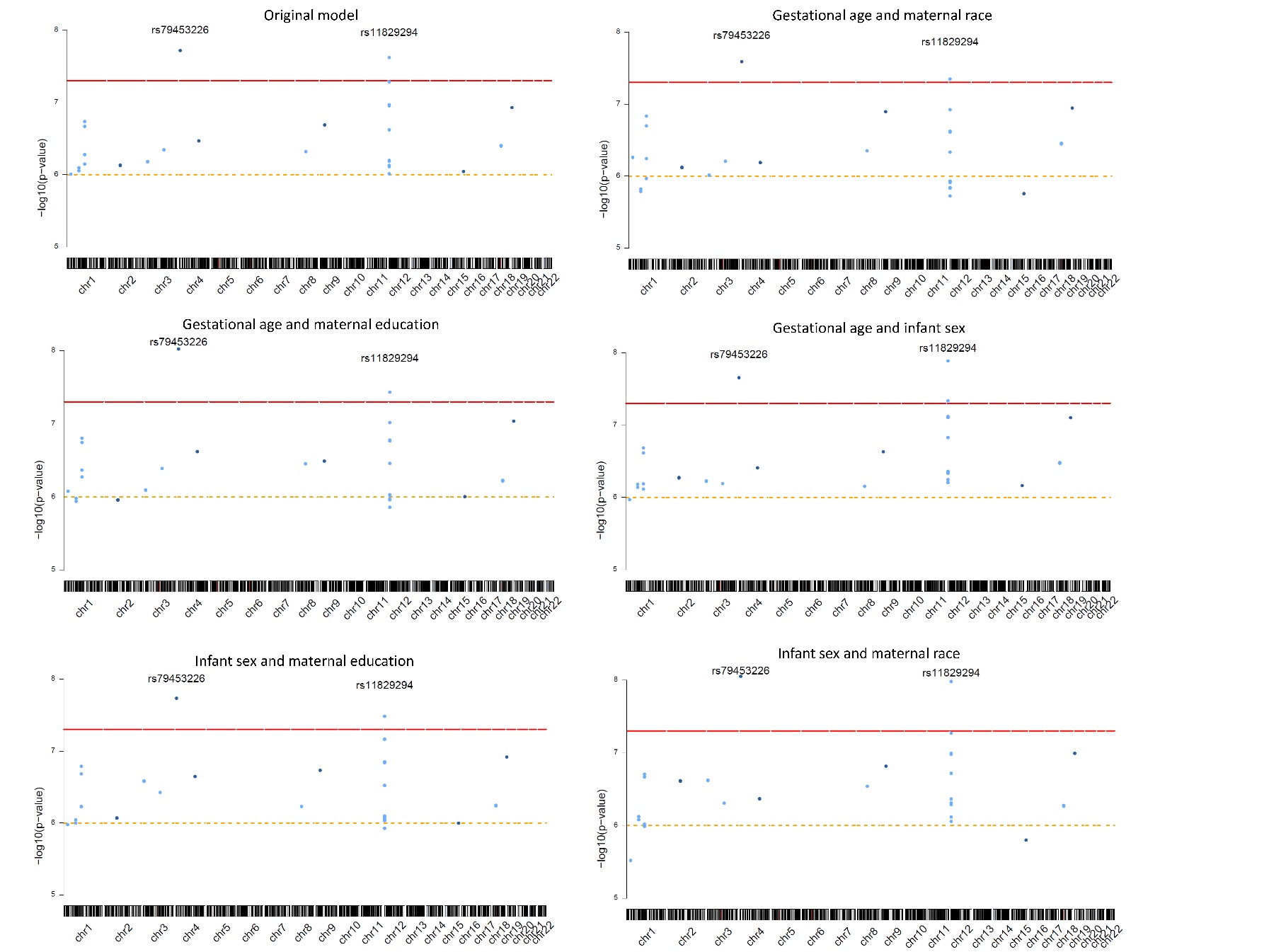
Fig. S2 Manhattan plots.** Each Manhattan plot shows results for single variant association testing with a different set of covariates. The title of each plot indicates whether it was the original model with no interaction terms or which interaction terms were included. The Manhattan plot visualizes the association, with the LPAx trait, of suggestive SNPs identified in the original model along the genome. X-axis represents genomic location and y-axis represents -log10(p-value). Each dot represents a SNP tested. SNPs above the red horizontal line, which marks p-value=5×10^-8^, are considered genome-wide significant. SNPs above the yellow dashed line, which marks p-value=1×10^-6^, are considered suggestive. This plot was generated using the R package *karyoploteR* [3]. NCBI build 38.

**References**

1. Yang J, Benyamin B, McEvoy BP, Gordon S, Henders AK, Nyholt DR, et al. Common SNPs explain a large proportion of the heritability for human height. Nat. Genet. 2010;42:565–569.

2. Zaitlen N, Kraft P, Patterson N, Pasaniuc B, Bhatia G, Pollack S, et al. Using extended genealogy to estimate components of heritability for 23 quantitative and dichotomous traits. PLoS Genet. 2013;9:e1003520.

3. Gel B, Serra E. karyoploteR: an R/Bioconductor package to plot customizable genomes displaying arbitrary data. Bioinformatics. 2017;33:3088–3090.
